# *N*′-(4-Methoxy­benzyl­idene)-4-nitro­benzo­hydrazide methanol solvate

**DOI:** 10.1107/S1600536808005813

**Published:** 2008-03-05

**Authors:** Yuan-Zhi Wang, Ming-Dong Wang, Yun-Peng Diao, Qian Cai

**Affiliations:** a Liaoning University of Traditional Chinese Medicine, Shenyang 110032, People’s Republic of China; b Liaoning Food and Drug Administration, Shenyang 110003, People’s Republic of China; c School of Pharmacy, Dalian Medical University, Dalian 116044, People’s Republic of China

## Abstract

The title compound, C_15_H_13_N_3_O_4_·CH_4_O, was synthesized from the reaction of 4-methoxy­benzaldehyde with 4-nitro­benzohydrazide in methanol. The benzene rings of the Schiff base mol­ecule are nearly coplanar, making a dihedral angle of 7.0 (3)°. The methanol solvent mol­ecules are linked to the Schiff base mol­ecules by N—H⋯O, O—H⋯N and O—H⋯O hydrogen bonds, forming chains running parallel to the *b* axis.

## Related literature

For related structures, see: Brückner *et al.* (2000 ▶); Diao (2007 ▶); Diao *et al.* (2007 ▶, 2008 ▶); Harrop *et al.* (2003 ▶); Huang *et al.* (2007 ▶); Li *et al.* (2007 ▶); Ren *et al.* (2002 ▶).
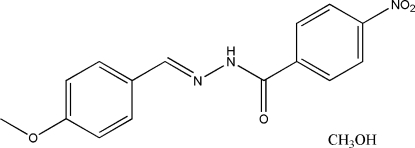


## Experimental

### 

#### Crystal data

C_15_H_13_N_3_O_4_·CH_4_O
*M* *_r_* = 331.33
Monoclinic, 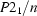

*a* = 14.719 (3) Å
*b* = 6.631 (2) Å
*c* = 18.002 (3) Å
β = 113.17 (3)°
*V* = 1615.3 (7) Å^3^
*Z* = 4
Mo *K*α radiation
μ = 0.10 mm^−1^
*T* = 298 (2) K
0.27 × 0.23 × 0.23 mm

#### Data collection

Bruker SMART CCD area-detector diffractometer
Absorption correction: multi-scan (*SADABS*; Bruker, 2000 ▶) *T*_min_ = 0.973, *T*_max_ = 0.977
9171 measured reflections
3351 independent reflections
1493 reflections with *I* > 2σ(*I*)
*R*_int_ = 0.065

#### Refinement

*R*[*F* ^2^ > 2σ(*F* ^2^)] = 0.057
*wR*(*F* ^2^) = 0.172
*S* = 0.95
3351 reflections
224 parameters
1 restraint
H atoms treated by a mixture of independent and constrained refinement
Δρ_max_ = 0.17 e Å^−3^
Δρ_min_ = −0.17 e Å^−3^

### 

Data collection: *SMART* (Bruker, 2000 ▶); cell refinement: *SAINT* (Bruker, 2000 ▶); data reduction: *SAINT*; program(s) used to solve structure: *SHELXTL* (Sheldrick, 2008 ▶); program(s) used to refine structure: *SHELXTL*; molecular graphics: *SHELXTL*; software used to prepare material for publication: *SHELXTL*.

## Supplementary Material

Crystal structure: contains datablocks global, I. DOI: 10.1107/S1600536808005813/rz2199sup1.cif

Structure factors: contains datablocks I. DOI: 10.1107/S1600536808005813/rz2199Isup2.hkl

Additional supplementary materials:  crystallographic information; 3D view; checkCIF report

## Figures and Tables

**Table 1:** Hydrogen-bond geometry (Å, °)

*D*—H⋯*A*	*D*—H	H⋯*A*	*D*⋯*A*	*D*—H⋯*A*
N2—H2*A*⋯O5	0.897 (10)	2.049 (13)	2.921 (3)	164 (3)
O5—H5⋯N3^i^	0.82	2.56	3.167 (3)	133
O5—H5⋯O3^i^	0.82	2.10	2.863 (3)	154

Symmetry code: (i) 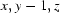.

**Fig. 1.**
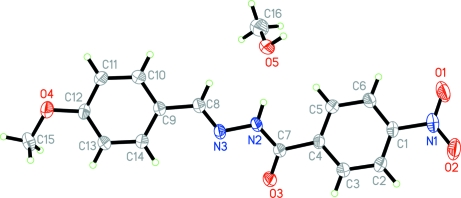
The structure of the title compound with displacement ellipsoids drawn at the 30% probability level.

## supplementary crystallographic information

### Comment

Schiff base compounds have been found to have potential pharmacological and antitumor properties (Brückner *et al.*, 2000; Harrop *et al.*, 2003; Ren *et al.*, 2002). Recently, a few Schiff base compounds derived from the reaction of aldehydes with benzohydrazides have been reported (Diao *et al.*, 2008; Diao *et al.*, 2007; Diao, 2007; Li *et al.*, 2007; Huang *et al.*, 2007). As a further study of such compounds, we report here the structure of the title compound.

The title compound (Fig. 1) consists of a Schiff base molecule and a lattice methanol molecule. The Schiff base molecule is nearly planar with the dihedral angle between the two phenyl rings of 7.0 (3)°. The dihedral angle between the C1—C6 phenyl ring and the O1/N1/O2 nitryl plane is 5.1 (3)°. The torsion angles C9—C8—N3—N2 and C4—C7—N2—N3 are 1.4 (3) and 1.6 (3)°, respectively. The methanol molecules are linked to the Schiff base molecules by N–H···O, O–H···N and O–H···O hydrogen bonds (Table 1) forming chains running parallel to the *b* axis.

### Experimental

4-Methoxybenzaldehyde (0.1 mmol, 13.6 mg) and 4-nitrobenzohydrazide (0.1 mmol, 18.1 mg) were dissolved in methanol (20 ml). The mixture was stirred at reflux for 1 h and cooled to room temperature. After keeping the solution in air for five days, yellow block-like crystals were formed on slow evaporation of the solvent.

### Refinement

H2A was located from a difference Fourier map and refined isotropically, with the N–H distance restrained to 0.90 (1) Å. Other H atoms were placed in calculated positions and constrained to ride on their parent atoms, with C–H distances of 0.93–0.96 Å, O–H distance of 0.82 Å, and with *U*_iso_(H) = 1.2*U*_eq_(C) and 1.5*U*_eq_(O and methyl C).

### Crystal data

**Table:**

C_15_H_13_N_3_O_4_·CH_4_O	*F*_000_ = 696
*M**_r_* = 331.33	*D*_x_ = 1.362 Mg m^−^^3^
Monoclinic, *P*2_1_/*n*	Mo *K*α radiation λ = 0.71073 Å
Hall symbol: -P 2yn	Cell parameters from 670 reflections
*a* = 14.719 (3) Å	θ = 2.4–24.5º
*b* = 6.631 (2) Å	µ = 0.10 mm^−^^1^
*c* = 18.002 (3) Å	*T* = 298 (2) K
β = 113.17 (3)º	Block, yellow
*V* = 1615.3 (7) Å^3^	0.27 × 0.23 × 0.23 mm
*Z* = 4	

### Data collection

**Table:**

Bruker SMART CCD area-detector diffractometer	3351 independent reflections
Radiation source: fine-focus sealed tube	1493 reflections with *I* > 2σ(*I*)
Monochromator: graphite	*R*_int_ = 0.065
*T* = 298(2) K	θ_max_ = 26.5º
ω scans	θ_min_ = 1.5º
Absorption correction: multi-scan(SADABS; Bruker, 2000)	*h* = −18→18
*T*_min_ = 0.973, *T*_max_ = 0.977	*k* = −8→7
9171 measured reflections	*l* = −17→22

### Refinement

**Table:**

Refinement on *F*^2^	Secondary atom site location: difference Fourier map
Least-squares matrix: full	Hydrogen site location: inferred from neighbouring sites
*R*[*F*^2^ > 2σ(*F*^2^)] = 0.057	H atoms treated by a mixture of independent and constrained refinement
*wR*(*F*^2^) = 0.172	*w* = 1/[σ^2^(*F*_o_^2^) + (0.07*P*)^2^] where *P* = (*F*_o_^2^ + 2*F*_c_^2^)/3
*S* = 0.95	(Δ/σ)_max_ < 0.001
3351 reflections	Δρ_max_ = 0.17 e Å^−^^3^
224 parameters	Δρ_min_ = −0.17 e Å^−^^3^
1 restraint	Extinction correction: SHELXTL (Sheldrick, 2008), Fc^*^=kFc[1+0.001xFc^2^λ^3^/sin(2θ)]^-1/4^
Primary atom site location: structure-invariant direct methods	Extinction coefficient: 0.0051 (12)

### Special details

**Table:**

Geometry. All e.s.d.'s (except the e.s.d. in the dihedral angle between two l.s. planes) are estimated using the full covariance matrix. The cell e.s.d.'s are taken into account individually in the estimation of e.s.d.'s in distances, angles and torsion angles; correlations between e.s.d.'s in cell parameters are only used when they are defined by crystal symmetry. An approximate (isotropic) treatment of cell e.s.d.'s is used for estimating e.s.d.'s involving l.s. planes.
Refinement. Refinement of *F*^2^ against ALL reflections. The weighted *R*-factor *wR* and goodness of fit *S* are based on *F*^2^, conventional *R*-factors *R* are based on *F*, with *F* set to zero for negative *F*^2^. The threshold expression of *F*^2^ > σ(*F*^2^) is used only for calculating *R*-factors(gt) *etc*. and is not relevant to the choice of reflections for refinement. *R*-factors based on *F*^2^ are statistically about twice as large as those based on *F*, and *R*- factors based on ALL data will be even larger.

### Fractional atomic coordinates and isotropic or equivalent isotropic displacement parameters (Å^2^)

**Table:**

	*x*	*y*	*z*	*U*_iso_*/*U*_eq_	
N1	0.3893 (2)	0.1141 (5)	0.43054 (18)	0.0628 (8)	
N2	0.37993 (17)	0.4842 (3)	0.09474 (14)	0.0480 (6)	
N3	0.38196 (16)	0.5979 (3)	0.03071 (14)	0.0481 (6)	
O1	0.35466 (19)	−0.0546 (4)	0.41861 (15)	0.0913 (9)	
O2	0.4269 (2)	0.1901 (4)	0.49761 (15)	0.0915 (9)	
O3	0.38954 (18)	0.7649 (3)	0.16870 (13)	0.0712 (7)	
O4	0.37929 (16)	0.8238 (3)	−0.31725 (12)	0.0643 (6)	
O5	0.34226 (17)	0.0632 (3)	0.04415 (13)	0.0643 (6)	
H5	0.3646	−0.0390	0.0708	0.096*	
C1	0.3883 (2)	0.2315 (4)	0.36036 (17)	0.0474 (8)	
C2	0.4211 (2)	0.4269 (5)	0.37219 (18)	0.0587 (8)	
H2	0.4442	0.4847	0.4234	0.070*	
C3	0.4188 (2)	0.5348 (5)	0.30657 (18)	0.0570 (8)	
H3	0.4402	0.6680	0.3136	0.068*	
C4	0.38535 (19)	0.4503 (4)	0.23005 (16)	0.0433 (7)	
C5	0.3527 (2)	0.2511 (4)	0.22030 (18)	0.0500 (8)	
H5A	0.3303	0.1916	0.1695	0.060*	
C6	0.3537 (2)	0.1412 (4)	0.28616 (18)	0.0520 (8)	
H6	0.3311	0.0088	0.2799	0.062*	
C7	0.3851 (2)	0.5799 (4)	0.16214 (17)	0.0484 (7)	
C8	0.3756 (2)	0.4971 (4)	−0.03121 (18)	0.0492 (7)	
H8	0.3686	0.3578	−0.0305	0.059*	
C9	0.37877 (19)	0.5910 (4)	−0.10321 (16)	0.0432 (7)	
C10	0.3555 (2)	0.4751 (5)	−0.17273 (17)	0.0525 (8)	
H10	0.3393	0.3398	−0.1717	0.063*	
C11	0.3559 (2)	0.5571 (4)	−0.24324 (18)	0.0535 (8)	
H11	0.3389	0.4783	−0.2895	0.064*	
C12	0.3818 (2)	0.7574 (4)	−0.24426 (17)	0.0475 (7)	
C13	0.4075 (2)	0.8738 (4)	−0.17570 (17)	0.0497 (8)	
H13	0.4263	1.0075	−0.1762	0.060*	
C14	0.4054 (2)	0.7901 (4)	−0.10576 (17)	0.0495 (8)	
H14	0.4221	0.8695	−0.0597	0.059*	
C15	0.3969 (2)	1.0303 (5)	−0.3251 (2)	0.0705 (10)	
H15A	0.3480	1.1096	−0.3156	0.106*	
H15B	0.3933	1.0560	−0.3787	0.106*	
H15C	0.4614	1.0657	−0.2864	0.106*	
C16	0.2426 (3)	0.0315 (5)	−0.0070 (2)	0.0854 (11)	
H16A	0.2053	0.0016	0.0250	0.128*	
H16B	0.2380	−0.0796	−0.0425	0.128*	
H16C	0.2166	0.1508	−0.0383	0.128*	
H2A	0.377 (2)	0.3496 (16)	0.0892 (19)	0.080*	

### Atomic displacement parameters (Å^2^)

**Table:**

	*U*^11^	*U*^22^	*U*^33^	*U*^12^	*U*^13^	*U*^23^
N1	0.0707 (18)	0.072 (2)	0.0519 (19)	0.0074 (16)	0.0308 (16)	0.0221 (16)
N2	0.0678 (16)	0.0380 (13)	0.0453 (15)	−0.0013 (12)	0.0298 (13)	0.0086 (12)
N3	0.0634 (15)	0.0436 (14)	0.0426 (15)	0.0012 (12)	0.0266 (13)	0.0089 (12)
O1	0.1079 (19)	0.0897 (19)	0.0742 (18)	−0.0230 (16)	0.0335 (15)	0.0321 (15)
O2	0.137 (2)	0.096 (2)	0.0475 (16)	0.0115 (16)	0.0432 (16)	0.0156 (15)
O3	0.128 (2)	0.0367 (12)	0.0565 (14)	−0.0021 (12)	0.0437 (14)	0.0032 (10)
O4	0.0934 (16)	0.0610 (14)	0.0450 (13)	−0.0019 (12)	0.0342 (12)	0.0065 (11)
O5	0.0883 (16)	0.0432 (13)	0.0554 (15)	−0.0041 (12)	0.0218 (13)	0.0050 (10)
C1	0.0540 (18)	0.0532 (19)	0.0393 (18)	0.0052 (15)	0.0230 (15)	0.0101 (15)
C2	0.081 (2)	0.060 (2)	0.0378 (18)	−0.0057 (18)	0.0263 (16)	−0.0037 (15)
C3	0.080 (2)	0.0474 (18)	0.0464 (19)	−0.0068 (16)	0.0277 (17)	0.0011 (15)
C4	0.0526 (17)	0.0442 (17)	0.0360 (17)	0.0037 (14)	0.0208 (14)	0.0049 (13)
C5	0.0615 (19)	0.0465 (18)	0.0440 (18)	−0.0040 (15)	0.0229 (16)	−0.0025 (14)
C6	0.0664 (19)	0.0466 (18)	0.050 (2)	−0.0042 (15)	0.0306 (16)	0.0039 (15)
C7	0.0634 (19)	0.0404 (18)	0.0434 (18)	−0.0016 (15)	0.0231 (16)	0.0035 (15)
C8	0.0605 (19)	0.0452 (17)	0.0458 (18)	−0.0016 (15)	0.0253 (16)	0.0052 (15)
C9	0.0489 (16)	0.0420 (16)	0.0404 (17)	−0.0004 (14)	0.0194 (14)	0.0058 (13)
C10	0.0657 (19)	0.0420 (17)	0.052 (2)	−0.0042 (14)	0.0247 (16)	−0.0003 (15)
C11	0.072 (2)	0.0487 (19)	0.0405 (18)	−0.0036 (16)	0.0231 (16)	−0.0042 (15)
C12	0.0551 (18)	0.0543 (19)	0.0362 (17)	0.0042 (15)	0.0214 (15)	0.0052 (15)
C13	0.0638 (19)	0.0410 (17)	0.0476 (18)	−0.0050 (14)	0.0254 (16)	0.0036 (14)
C14	0.0620 (19)	0.0472 (19)	0.0417 (18)	−0.0036 (15)	0.0231 (15)	0.0004 (14)
C15	0.087 (2)	0.066 (2)	0.067 (2)	−0.0062 (19)	0.040 (2)	0.0200 (18)
C16	0.092 (3)	0.075 (3)	0.087 (3)	−0.002 (2)	0.032 (2)	0.000 (2)

### Geometric parameters (Å, °)

**Table:**

N1—O1	1.213 (3)	C5—H5A	0.9300
N1—O2	1.222 (3)	C6—H6	0.9300
N1—C1	1.479 (4)	C8—C9	1.455 (4)
N2—C7	1.345 (3)	C8—H8	0.9300
N2—N3	1.388 (3)	C9—C14	1.383 (4)
N2—H2A	0.897 (10)	C9—C10	1.392 (4)
N3—C8	1.271 (3)	C10—C11	1.383 (4)
O3—C7	1.232 (3)	C10—H10	0.9300
O4—C12	1.372 (3)	C11—C12	1.384 (4)
O4—C15	1.411 (3)	C11—H11	0.9300
O5—C16	1.407 (4)	C12—C13	1.376 (4)
O5—H5	0.8200	C13—C14	1.388 (4)
C1—C6	1.366 (4)	C13—H13	0.9300
C1—C2	1.370 (4)	C14—H14	0.9300
C2—C3	1.370 (4)	C15—H15A	0.9600
C2—H2	0.9300	C15—H15B	0.9600
C3—C4	1.386 (4)	C15—H15C	0.9600
C3—H3	0.9300	C16—H16A	0.9600
C4—C5	1.392 (4)	C16—H16B	0.9600
C4—C7	1.493 (4)	C16—H16C	0.9600
C5—C6	1.387 (4)		
			
O1—N1—O2	123.4 (3)	C9—C8—H8	118.7
O1—N1—C1	118.2 (3)	C14—C9—C10	118.0 (3)
O2—N1—C1	118.4 (3)	C14—C9—C8	123.2 (3)
C7—N2—N3	118.7 (2)	C10—C9—C8	118.8 (3)
C7—N2—H2A	123 (2)	C11—C10—C9	121.3 (3)
N3—N2—H2A	118 (2)	C11—C10—H10	119.3
C8—N3—N2	115.1 (2)	C9—C10—H10	119.3
C12—O4—C15	118.0 (2)	C10—C11—C12	119.3 (3)
C16—O5—H5	109.5	C10—C11—H11	120.3
C6—C1—C2	122.6 (3)	C12—C11—H11	120.3
C6—C1—N1	118.6 (3)	O4—C12—C13	125.0 (3)
C2—C1—N1	118.7 (3)	O4—C12—C11	114.6 (3)
C1—C2—C3	118.2 (3)	C13—C12—C11	120.4 (3)
C1—C2—H2	120.9	C12—C13—C14	119.5 (3)
C3—C2—H2	120.9	C12—C13—H13	120.2
C2—C3—C4	121.6 (3)	C14—C13—H13	120.2
C2—C3—H3	119.2	C9—C14—C13	121.3 (3)
C4—C3—H3	119.2	C9—C14—H14	119.3
C3—C4—C5	118.6 (3)	C13—C14—H14	119.3
C3—C4—C7	117.8 (3)	O4—C15—H15A	109.5
C5—C4—C7	123.6 (3)	O4—C15—H15B	109.5
C6—C5—C4	120.2 (3)	H15A—C15—H15B	109.5
C6—C5—H5A	119.9	O4—C15—H15C	109.5
C4—C5—H5A	119.9	H15A—C15—H15C	109.5
C1—C6—C5	118.7 (3)	H15B—C15—H15C	109.5
C1—C6—H6	120.7	O5—C16—H16A	109.5
C5—C6—H6	120.7	O5—C16—H16B	109.5
O3—C7—N2	122.6 (3)	H16A—C16—H16B	109.5
O3—C7—C4	120.8 (3)	O5—C16—H16C	109.5
N2—C7—C4	116.6 (2)	H16A—C16—H16C	109.5
N3—C8—C9	122.6 (3)	H16B—C16—H16C	109.5
N3—C8—H8	118.7		

### Hydrogen-bond geometry (Å, °)

**Table:**

*D*—H···*A*	*D*—H	H···*A*	*D*···*A*	*D*—H···*A*
N2—H2A···O5	0.897 (10)	2.049 (13)	2.921 (3)	164 (3)
O5—H5···N3^i^	0.82	2.56	3.167 (3)	133
O5—H5···O3^i^	0.82	2.10	2.863 (3)	154

Symmetry codes: (i) *x*, *y*−1, *z*.
